# Transcriptome sequencing reveals differences between leydig cells and sertoli cells of yak

**DOI:** 10.3389/fvets.2022.960250

**Published:** 2022-08-24

**Authors:** Yaying Wang, Yangyang Pan, Meng Wang, Seth Yaw Afedo, Ling Zhao, Xiaohong Han, Minqing Liu, Tian Zhao, Tongxiang Zhang, Tianyi Ding, Jinglei Wang, Yan Cui, Sijiu Yu

**Affiliations:** ^1^College of Veterinary Medicine, Gansu Agricultural University, Lanzhou, China; ^2^Gansu Province Livestock Embryo Engineering Research Center, Lanzhou, China

**Keywords:** yak, testis, LCs, SCs, RNA sequence, differential analysis

## Abstract

In this study, we detected the expression of mRNAs, lncRNAs, and miRNAs in primary cultured leydig cells (LCs) and sertoli cells (SCs) of yak by RNA sequencing technology. A total of 84 differently expression mRNAs (DEmRNAs) (LCs vs. SCs: 15 up and 69 down), 172 differently expression lncRNAs (DElncRNAs) (LCs vs. SCs: 36 up and 136 down), and 90 differently expression miRNAs (DEmiRNAs) (LCs vs. SCs: 72 up and 18 down) were obtained between the two types of cells. GO enrichment and KEGG analysis indicated that the differential expression genes (DEGs) were more enriched in the regulation of actin cytoskeleton, Rap1/MAPK signaling pathway, steroid biosynthesis, focal adhesion, and pathways associated with metabolism. Targeted regulation relationship pairs of 3β-HSD and MSTRG.54630.1, CNTLN and MSTRG.19058.1, BRCA2 and MSTRG.28299.4, CA2 and novel-miR-148, and ceRNA network of LAMC3-MSTRG.68870.1- bta-miR-7862/novel-miR-151/novel-miR-148 were constructed by Cytoscape software. In conclusion, the differences between LCs and SCs were mainly reflected in steroid hormone synthesis, cell proliferation and metabolism, and blood-testicular barrier (BTB) dynamic regulation, and 3β-HSD, CNTLN, BRCA2, CA2, and LAMC3 may be the key factors causing these differences, which may be regulated by ncRNAs. This study provides a basic direction for exploring the differential regulation of LCs and SCs by ncRNAs.

## Introduction

Yaks live in high altitudes more than 3,000 meters above sea level and are important species in these low-temperature ecosystems with extreme environmental conditions such as thin air, strong ultraviolet rays, lack of forage resources, and low temperature most of the year. They are the main source of livelihood for agro-pastoralists as they are used to produce milk, meat, and other derived products. However, the natural fertility of yaks is very low, and they usually give birth to one calf in 2 years or two calves in 3 years. Therefore, we are committed to exploring the reproductive regulation mechanism of yak to lay a strong foundation for yak breeding and industry expansion.

The testis is an important internal reproductive organ of male, which produces sperms and secretes androgen to maintain male fertility and masculinity. Spermatogenesis is a continuous, rigorous, and multifactor regulated process that includes self-renewal and differentiation of spermatogonial stem cells (SSCs), meiosis of spermatocytes, and spermiogenesis (1). Sertoli cells (SCs), regarded as “nurse cells,” are the only type of somatic cells that are in direct contact with germ cells (GCs), and play a major role in spermatogenesis by facilitating the development, proliferation, and differentiation of GCs. SCs constitute the blood–testicular barrier (BTB) which divides seminiferous tubules into basal and apical sections by tight junctions, gap junctions, and adherent junctions, and is the basis of testicular immune privilege that insulates late-stage GCs from the body's immune system (2). Leydig cells (LCs), located in the loose connective tissue between the seminiferous tubules, synthesize and secrete most of the body's androgens. Testosterone is one of the principal and representative androgens in mammals; it regulates the progression of spermatogenesis by identifying and binding to androgen receptors on SCs. Without the stimulation of testosterone or knockout of androgen receptors, the integrity of the BTB is impaired, and GCs stop differentiating and are phagocytosed by SCs, at the same time, while fully mature spermatozoa cannot be released from SCs (3). Studies on humans have shown that men with higher testosterone levels are more likely to marry (4), and endogenous testosterone was positively associated with thromboembolism, heart failure, and myocardial infarction in men (5). A complex network of signal exchanges between LCs and SCs in the testis controls embryo development and male gamete production. A recent study has demonstrated that paracrine factors secreted by the testicular microenvironment composed of SCs and peritubular myoid cells regulate the endogenous function of LCs by modulating the hedgehog signaling pathway (6). In addition, the transfer of miRNAs that depend on SC-derived exosomes inhibits LC steroidogenesis by targeting steroidogenic factor 1 (7).

RNA sequencing has widely been used in animals to mine potential regulatory molecules for many years. For instance, using this approach, researchers have identified several pathways by which *KLF6* is involved in lipid metabolism (8). They also discovered that polymorphisms of *LEPR* are associated with fat deposition, while polymorphisms *of PLIN1* are associated with carcass quality and body measurement traits in beef cattle by analyzing their gene sequences and identification of SNPs (9, 10). Single-cell sequencing has revealed that the mesenchymal stromal population is involved in the regeneration of adult LCs and that the proliferation and differentiation of stem LCs are regulated separately, because proliferating and differentiating groups of cells specifically express certain genes (11). Non-coding RNAs (ncRNAs) are important members of intracellular gene regulatory networks, and their epigenetic regulation is a research hotspot in current biological fields. Long non-coding RNAs (lncRNAs), with multiple exons and more than 200 nucleotides, play a key regulatory role in a variety of biological processes by functioning as a molecular bait, molecular signal, and molecular scaffold in the body through molecular guidance (12). microRNAs (miRNAs) are a class of endogenous short (20–25nt) ncRNAs, that can regulate gene expression in the phase of transcriptional or post-transcriptional regulation by targeting the 3′-untranslated region and/or coding region of messenger RNAs (mRNAs). Studies show that lncRNAs and miRNAs are involved in the differentiation of gonads, spermatogonial maintenance, proliferation, and differentiation of spermatocytes, spermatogenesis, maturation and viability of spermatozoa in epididymis, and GCs apoptosis in mammals (13–15). miRNAs also participate in the homeostasis regulation of sheep testicular immune privilege by regulating immune-related genes to maintain BTB function and inhibit immune response under normal physiological conditions (16).

Although a large number of lncRNAs and miRNAs have been identified and demonstrated to be involved in mammalian testicular function regulation (17–19), there are few studies on the expression of ncRNAs in different cells of the testis, especially on their regulatory mechanisms. It is valuable to study which non-coding genes maintain the normal physiological function of the testis by targeting LCs and SCs. In this study, next-generation sequencing technology was used to explore the differences in gene expression between LCs and SCs in yak testis, based on the research on the mechanism of cell regulation by ncRNAs.

## Materials and methods

### Ethical statement

This study was permitted by the Ethics Committee of Gansu Agricultural University, China (Ethic approval file No. GSAU-Eth-VMC-2022-19). Animal experimentation, including sample collection, was performed in agreement with the guidelines of the ethical committee of Gansu Agricultural University. Furthermore, the experimental protocol complied with the local animal welfare guidelines.

### Isolation and culture of yak testicular cells

Three healthy 1-year-old male yak testicles were harvested, washed, and placed in a 0.9% NaCl solution containing 1% penicillin and streptomycin. The sheath was peeled off, and physiological saline was injected into the testicular arteries to flush the blood vessels and reduce the contamination of red blood cells. Then the samples were transferred to an ultra-clean table, where outer white membranes were peeled off, a piece of testicular tissue was cut out, placed in a sterile dish, and two tweezers were used to make the testicular tissue lose. With reference to methods reported by Klinefelter et al. (20) with a simple modification, we isolated and purified LCs and SCs of yak testis by enzymatic digestion, gradient centrifugation, differential adherence, starvation culture, hypotonic treatment, etc. The loose testicular tissue mass was transferred into a conical flask with 0.1% collagenase I + 0.03% hyaluronidase after washing with phosphate buffer solution (PBS), then placed in a 37°C constant temperature and after which it was placed in a water bath for digestion for 30 min. The mixture was filtered successively through 100, 200, and 400 mesh filters to separate fully dispersed seminiferous tubules and individual interstitial cells. In order to obtain LCs, individual interstitial cells in the filtrate were washed with PBS and resuspended in DMEM/F-12, then added into a prepared Percoll gradient centrifuge (the densities of Percoll were 1.090, 1.080, 1.065, and 1.045 g/mL) gently, and centrifuged at 1,000 × g at 4°C for 20 min. Cells in the Percoll of 1.080 g/mL were collected and washed with PBS, then placed in the cell culture flask at 10^5^/mL with DMEM/F-12 containing 10% FBS. The cells were cultured in a 37°C humid chamber with 5% CO_2_. In order to obtain SCs, dispersed seminiferous tubules that were blocked by the filter screen were digested with 0.25% trypsin at 37°C and placed in a water bath for 40 min for digestion. After terminating the digestion with a serum-containing medium, the suspension was filtered successively with 100, 200, and 400 mesh filters. The collected cells were washed with PBS and cultured at 5 × 10^6^/mL with DMEM/F-12 containing 1% fetal bovine serum (FBS) in a 37°C humid chamber with 5% CO_2_. Five hours later, the medium was replaced with a serum-free medium. After 4 days of starvation culture, cells were treated with a hypotonic solution composed of 20 mM Tris–HCl (pH 7.4) for 5 min and the media was changed to DMEM/F-12 containing 5% FBS. When cells grew to 90% confluence, they were collected for subsequent study.

### Identification of yak testicular cells

#### 3β-HSD stain

3β-HSD is an indispensable enzyme in the process of steroidogenesis. Detection of 3β-HSD activity can be used to identify testicular LCs. About 10 μL of cell suspension was pipetted evenly on the glass slide and allowed to dry completely. The cells were then treated with a fresh staining solution for 2 h, rinsed with distilled water, and observed under a microscope. The preparation of staining solution was in accordance with a previously reported method (20).

#### Feulgen stain

Feulgen stain was used to identify SCs. SCs were cultured on polylysine-coated cover glasses, and after growing to 90% confluence, they were fixed with 4% paraformaldehyde for 2 h. Feulgen staining kit (Solarbio, Beijing, China) was used to identify SCs according to the manufacturer's instructions.

#### Immunofluorescence stain

GATA4 is a transcription factor that can be used to identify SCs in testis (21). The fixed SCs were washed with PBS, treated with 0.1% triton-x (1 h) for permeabilization, and incubated with 2% bovine serum albumin (BSA) for 2 h to block protein non-specific binding, and the cells were incubated with primary antibody of rabbit anti-GATA4 (1:150 dilution, Bioss, China) and mouse anti-β-Tublin (1:150 dilution, Bioss, China) for 12 h at 4°C. Secondary antibodies of anti-rabbit IgG and anti-mouse IgG (1:1,000 dilution, CST, USA) were incubated for 2 h in a dark environment, and DAPI was used to stain the cell nucleus. The cells were washed with PBS three times in the middle of each step. The cell fluorescence was observed and photographic images were taken with a fluorescence microscope (Cytiva, USA). In this experiment, β-tublin was used to label cell morphology.

#### Testosterone test

To identify whether LCs have testosterone-producing ability, the cells of the second generation were cultured in flasks in three groups. After growing to 90% confluence, the media was replaced with a fresh one, then cell supernatant of three groups was collected at 24, 32, and 48 h, respectively, to detect testosterone concentration using bovine testosterone Elisa kit (Jianglai Biology, Shanghai, China). The culture media acted as a blank factor to remove errors in the serum. The detection sensitivity of the kit was 1.0 pg/mL, and the intra- and inter-assay coefficients of variation were less than 9 and 11%, respectively.

### RNA extraction, library construction, and RNA-seq

Total RNA of LCs and SCs of the second generation cultured *in vitro* were extracted from three independent experiments using instructions from TransZol (TransGen, Beijing, China) manual. The integrity and concentration of RNA were checked by gel electrophoresis and Agilent 2,100 Bioanalyzer (Agilent Technologies, Inc., Santa Clara, CA, USA). The linear RNAs were isolated by NEBNext Poly (A) mRNA Magnetic Isolation Module (NEB, E7490) that removed ribosomal RNA (rRNA). The cDNA library was constructed following the manufacturer's instructions of NEBNext Ultra RNA Library Prep Kit for Illumina (NEB, E7530) and NEBNext Multiplex Oligos for Illumina (NEB, E7500). In brief, the enriched linear RNAs were fragmented into approximately 200nt RNA inserts, which were reverse-transcribed to produce cDNA. The suitable fragments which were used to perform end-repair/dA-tail and adaptor ligation were isolated by Agencourt AMPure XP beads (Beckman Coulter, Inc.). miRNA library was constructed according to the manufacturer's NEBNext Ultra small RNA Sample Library Prep Kit (NEB, E7300) instructions. The T4 RNA Ligase 1 was ligated to the 3′ end of the RNA, and then T4 RNA Ligase 2 was ligated to the 5′ adapter. After that, the RNAs were synthesized into cDNA under reverse transcriptional reaction. Finally, the cDNA libraries of linear RNA and miRNA were sequenced on the Illumina HiSeq™ sequencing platform, respectively.

### Quality control and mapping to the reference genome

All the downstream analyses were based on high-quality clean data. Clean reads of linear RNA were obtained from raw data by removing reads containing adapter, ploy-N, and low-quality reads (such as unknown nucleotides > 5%). The clean reads were mapped to *bos mutus* (wild yak) genome (BosGru_v2.0) using the HISAT2 platform (22). String Tie software was used to assemble the transcriptome based on reads mapped to the reference genome (23). The unknown transcripts with lengths more than 200nt having more than two exons in linear RNA library were selected as lncRNA candidates and further screened using CPC2/CNCI/Pfam/CPAT which has the power to distinguish protein-coding genes from non-coding genes (24–27). Clean reads of miRNA were obtained by removing reads containing adapter, ploy-N, and low-quality reads from raw data, they were trimmed and cleaned by removing sequences smaller than 18nt or longer than 30nt. The reads for subsequent analysis were obtained by using Bowtie tools (28) to filter out ribosomal RNA (rRNA), transfer RNA (tRNA), small nuclear RNA (snRNA), small nucleolar RNA (snoRNA), and other ncRNAs which aligned with the databases of Silva, GtRNAdb, Rfam, and Repbase from clean reads. The database of miRbase and miRDeep2 (29) were used to identify and predict miRNAs.

Gene abundance differences of linear RNA and miRNA between LCs and SCs were calculated based on the ratio of FPKM and TPM with the DESeq2 method, respectively (30–32).

### Validation of sequencing results by RT-qPCR

In order to confirm the accuracy of data from the RNA-seq approach, we randomly selected four mRNAs, six lncRNAs, and seven miRNAs to verify their expression level by RT-qPCR. About 0.8 μg total RNA was extracted and converted into cDNAs using Evo M-MLV RT Kit with gDNA Clean for qPCR II (Accurate Biology, Hunan, China) and Mir-X miRNA First-Strand Synthesis Kit (Takara, Tokyo, Japan), respectively, for detecting linear RNAs and miRNAs. PCR amplification experiment was carried out by TB Green® Premix Ex Taq™ (Takara, Tokyo, Japan). The amplification program of mRNAs and lncRNAs was as follows: 95°C for 5 min (Preheating), followed by 40 cycles of 95°C for 30 s (denaturation), 52–60°C for 20 s (annealing), and 72°C for 30 s (extension). The amplification program of miRNAs was as follows: 95°C for 5 min (Preheating), followed by 40 cycles of 95°C for 30 s (denaturation), and 60°C for 15 s (annealing). The primer sequences and annealing temperature are listed in Supplementary Table 1. β-actin and U6 were used as control genes of linear RNAs and miRNAs, respectively.

Genes' relative expression level of LCs vs. SCs was measured using the 2^−Δ*ΔCT*^ method. All data of RT-qPCR were analyzed with the software of IBM SPSS statistics 25.0 and were presented as mean values ± standard error of the mean (SEM) with three independent experiments (*n* = 3). Values of *P* < 0.05 were considered statistically significant.

### GO enrichment and KEGG pathway analysis

In order to get a better understanding of biological functions and potential mechanisms of ncRNA and mRNA between LCs and SCs, we performed Gene Ontology (GO) enrichment and Kyoto Encyclopedia of Genes and Genomes (KEGG) pathway analysis of differentially expressed genes (DEGs) and target genes of differentially expressed lncRNAs (DElncRNAs) and miRNAS (DEmiRNAs). The cis and trans target genes of lncRNAs were predicted according to positional relationship and expression level correlation between lncRNAs and mRNAs, respectively. The adjacent genes within the range of 100 kb of lncRNAs were considered cis target genes. Pearson's correlation coefficient method was used to analyze the correlation between lncRNAs and mRNAs among samples, and genes with absolute correlation > 0.9 and significant *p* < 0.01 were selected as trans target genes. The software miRanda and Targetscan were used to predict the target genes of miRNAs.

GO enrichment analysis was carried out using GOseq R packages based on Wallenius non-central hyper-geometric distribution. Statistical enrichment of the difference in KEGG pathways was analyzed with the KOBAS 2.0 software.

### Interaction network of DEmRNAs-DElncRNAs-DEmiRNAs

In order to further understand the regulatory role of lncRNAs and miRNAs on LCs and SCs, we constructed the network relationship of DEmRNAs-DElncRNAs, DEmRNAs-DEmiRNAs, and competing endogenous RNAs (ceRNAs) regulation network of DEmRNAs-DElncRNAs-DEmiRNAs using Cytoscape software, which is based on results prediction of lncRNAs and miRNAs target genes.

## Results

### Identification of yak testicular cells

**Figure 1:**
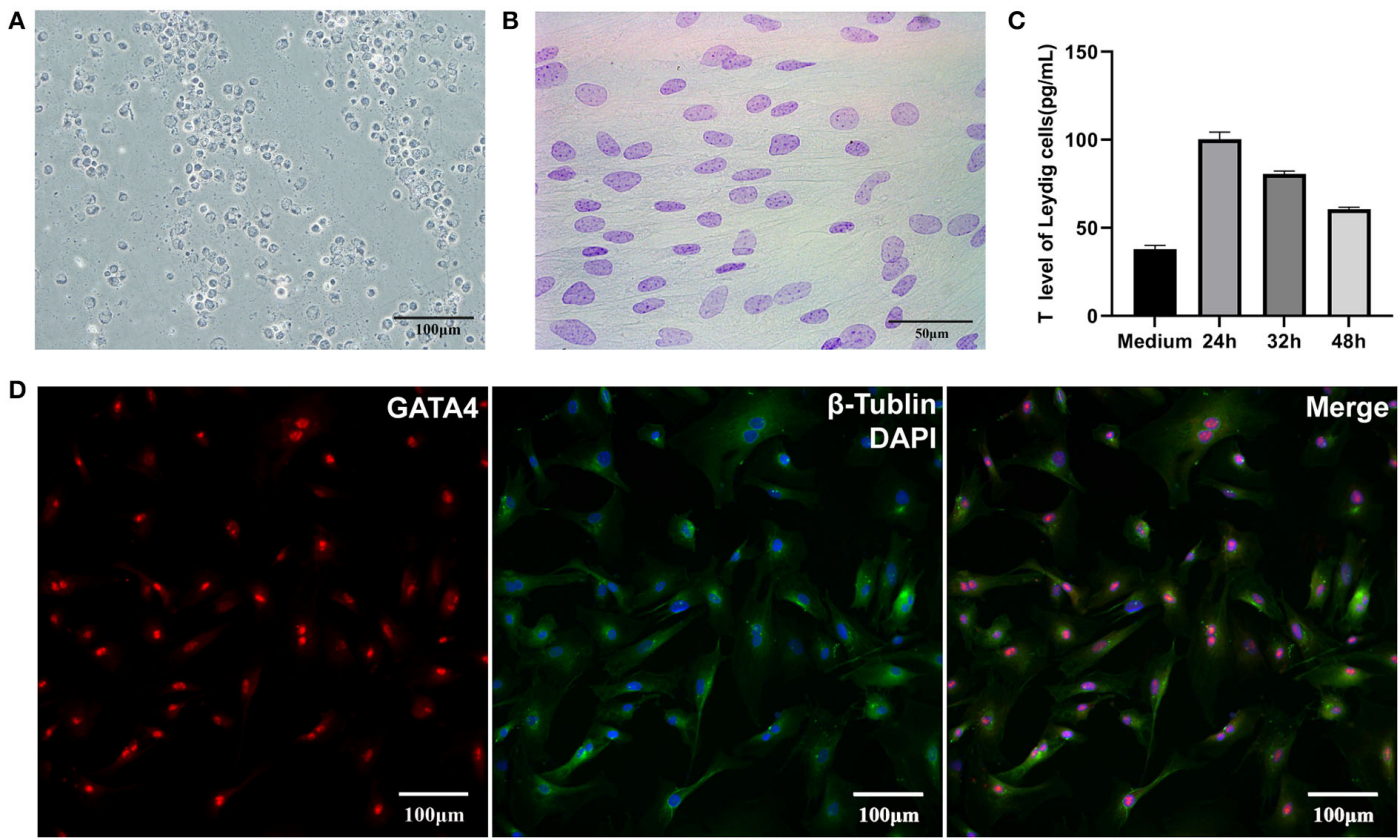
Identification of LCs and SCs. **(A)** 3β-HSD stained LCs, bar is 100 μm. **(B)** Feulgen-stained SCs, bar is 50 μm. **(C)** Testosterone secretion of LCs *in vitro*. **(D)**. GATA4 immunofluorescence stain of SCs, bar is 100 μm.

The results of 3β-HSD staining showed LCs (obtained through Percoll gradient centrifugation) as blue-purple with varying degrees, and this may be caused by the different enzyme activities in the cells (Figure 1A). The results of Feulgen staining showed the nucleus of SCs as purple-red with obvious dark particles, which appeared as “bipolar bodies,” also called “satellite bodies” (Figure 1B). Immunofluorescence staining with GATA4 showed that SCs from isolated tissues had strong fluorescence in their nucleus (Figure 1D). After identification, both LCs and SCs had high purity. ELISA results revealed that testosterone could be detected in culture media at 24, 32, and 48 h after cells have reached 90% confluence, but its concentration showed a downward trend, which might be caused by a high density of cells (Figure 1C). In short, the sub-cultured LCs still had the function of testosterone secretion.

### Sequencing results

**Figure 2:**
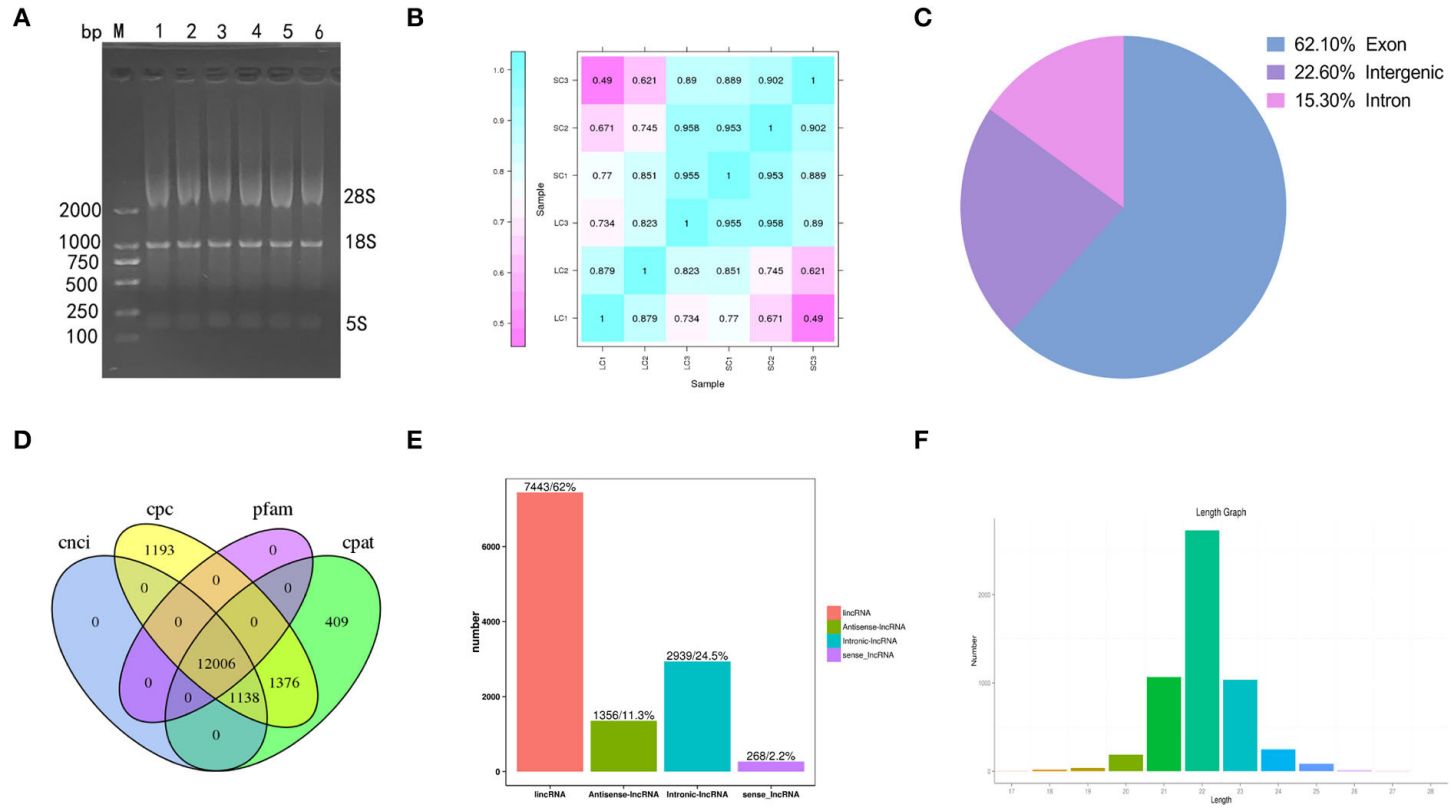
Results of sequencing. **(A)** Gel electrophoresis of total RNA. **(B)** Heat maps of correlation among samples. **(C)** The ratios of mapped reads are located at different positions in the reference genome. **(D)** The prediction of lncRNAs by CPC2/CNCI/Pfam/CPAT. **(E)** The number and proportion of lincRNAs, antisense-lncRNAs, intronic-lncRNAs, and sense-lncRNAs. **(F)** The length distribution of known miRNAs.

Gel electrophoresis of total RNA used for sequencing showed three bands of different sizes, indicating the integrity of the RNA (Figure 2A). The sample correlation heat map showed good repeatability between the three replicate samples (Figure 2B). A total of 119.91 Gb of clean data was obtained from the linear RNA library. The number of clean reads and Q30, mapped reads, and their ratio is shown in Table 1. The number of clean reads ranged from 17,454,472,800 to 23,140,535,100 among six samples, and Q30 was 93.47% and above. The mapped ratio ranged from 85.72 to 90.72%, obtained by aligning clean reads to the designated reference genome. And 62.1, 22.6, and 15.3% of mapped reads were located in the exons, intergenic regions, and intron regions, respectively (Figure 2C). By analyzing the mapped reads, a total of 5,767 genes were discovered, of which 2,774 genes had been annotated by using BLAST software to compare sequences to NR, Swiss-Prot, GO, COG, KEGG, and other databases (Table 2). A total of 12006 lncRNAs transcripts identified by CPC2/CNCI/Pfam/CPAT were taken for subsequent analysis (Figure 2D). There were four kinds of lncRNAs, namely, the number and proportion of lincRNAs, antisense-lncRNAs, intronic-lncRNAs, and sense-lncRNAs were 7,443 (62%), 1,356 (11.3%), 2,939 (24.5%), and 268 (2.2%), respectively (Figure 2E). A total of 162.24M clean reads were obtained by small RNA sequencing. As shown in Table 1, the clean reads in each sample were not less than 21,423,355, and Q30 and mapped ratios were greater than 96.75 and 63.72%, respectively. Then, a total of 6,312 miRNAs were obtained, of which 5,425 were known and 887 were novel miRNAs (Table 3). The length of most miRNAs was in the range of 21–23nt, and mainly 22nt, the picture shows the length distribution of known miRNAs (Figure 2F).

**Table 1:** Sequence results of six samples.

**Sample ID**	**Linear RNA**			**miRNA**		
	**Clean reads**	**Q30 (%)**	**Mapped reads (ratio)**	**Clean reads**	**Q30 (%)**	**Mapped reads (ratio)**
LC1	17,915,970,300	93.96	108,356, 619 (90.72%)	22,318,187	96.75	13,968,549 (63.72%)
LC2	17,454,472,800	93.47	104,655,013 (89.84%)	29,761,226	97.36	20,551,002 (70.35%)
LC3	23,140,535,100	93.80	138,189,142 (89.58%)	24,502,181	97.21	16,906,321 (70.14%)
SC1	22,442,633,700	93.82	131,263,482 (87.73%)	30,689,634	96.94	20,567,139 (68.55%)
SC2	19,244,031,600	93.63	112,358,805 (87.58%)	21,423,355	97.40	14,787,287 (70.19%)
SC3	19,720,968,900	93.86	112,697,605 (85.72%)	33,545,978	97.48	23,066,529 (70.26%)

**Table 2:** Function annotation result of new gene.

**Annotated databases**	**New gene number**
COG_Annotation	139
GO_Annotation	1,868
KEGG_Annotation	1,333
KOG_Annotation	728
Pfam_Annotation	541
Swissprot_Annotation	693
eggNOG_Annotation	1,738
nr_Annotation	2,752
All_Annotated	2,774

**Table 3:** Statistical results of miRNA.

**Sample**	**Known miRNAs**	**Novel miRNAs**	**Total**
LC1	4,960	680	5,640
LC2	5,066	735	5,801
LC3	5,059	736	5,795
SC1	5,116	777	5,893
SC2	4,954	661	5,615
SC3	5,082	785	5,867
Total	5,425	887	6,312

**Figure 3:**
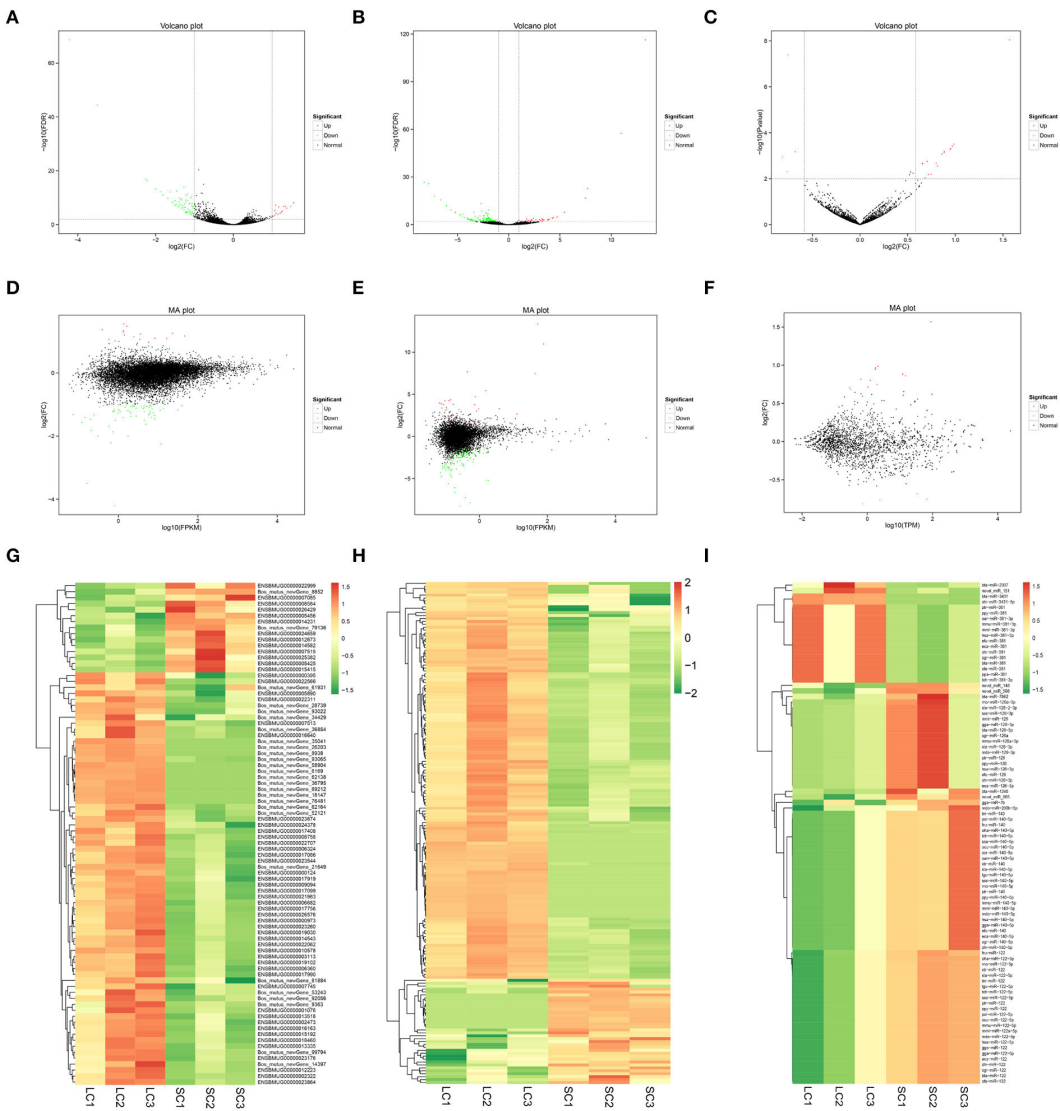
Volcano map, MA plot, and hierarchical clustering heat maps of DEmRNAs, DElncRNAs, and DEmiRNAs. **(A)** Volcano map of DEmRNAs. **(B)** Volcano map of DElncRNAs. **(C)** Volcano map of DEmiRNAs. **(D)** MA plot of DEmRNAs. **(E)** MA plot of DElncRNAs. **(F)** MA plot of DEmiRNAs. **(G)** Heat maps of DEmRNAs. **(H)** Heat maps of DElncRNAs. **(I)** Heat maps of DEmiRNAs.

Eighty-four DEGs were identified, of which 15 genes were upregulated and 69 genes downregulated (fold change ≥ 2 and FDR < 0.01) (Supplementary Table 2). A total of 172 DElncRNAs were found between LCs and SCs, of which 36 genes were upregulated and 136 genes downregulated (fold change ≥ 2 and FDR < 0.01) (Supplementary Table 3). At the same time, there were 90 miRNAs to be admitted as DEmiRNAs, of which 72 were upregulated and 18 downregulated (fold change ≥ 1.5 and *P* ≤ 0.01) (Supplementary Table 4). All differentially expressed RNAs between LCs and SCs are shown directly by the volcano map (Figures 3A–C), and the overall distribution of expression level and differential expression of genes between the two groups could be visually viewed by the MA plot (Figures 3D–F). Hierarchical clustering analysis based on expression patterns was performed and heatmaps are shown in Figures 3G–I.

### Validation of RNA-seq results by RT-qPCR

**Figure 4:**
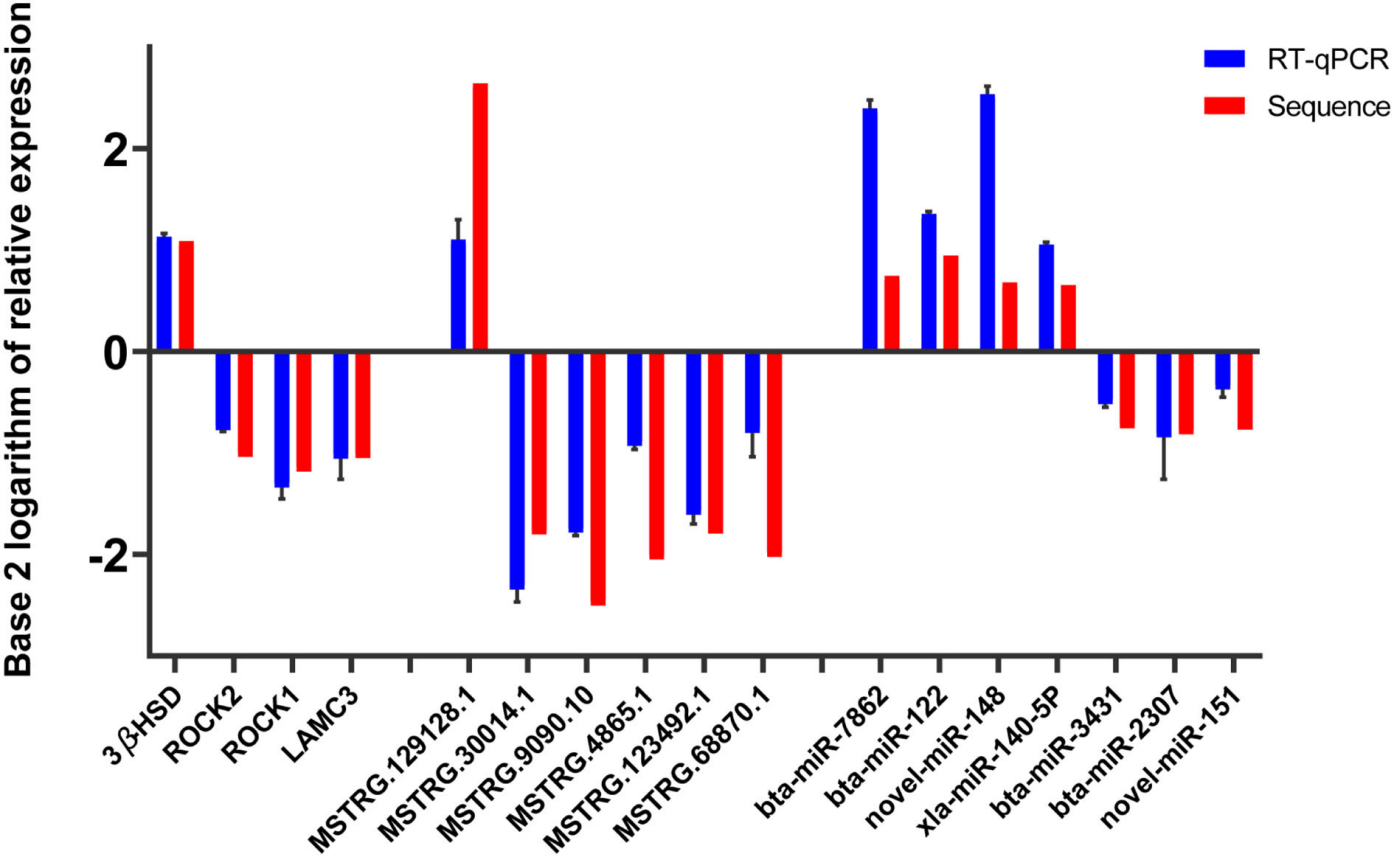
Validation of RT-qPCR.

The base 2 logarithms of 2^−Δ*ΔCT*^ and fold change obtained by sequencing were taken to draw a histogram, and the comparative results were as follows (Figure 4). The data from the two methods showed similar expression trends, proving the reliability of the RNA sequence data.

### GO and KEGG analysis of DEGs

**Figure 5:**
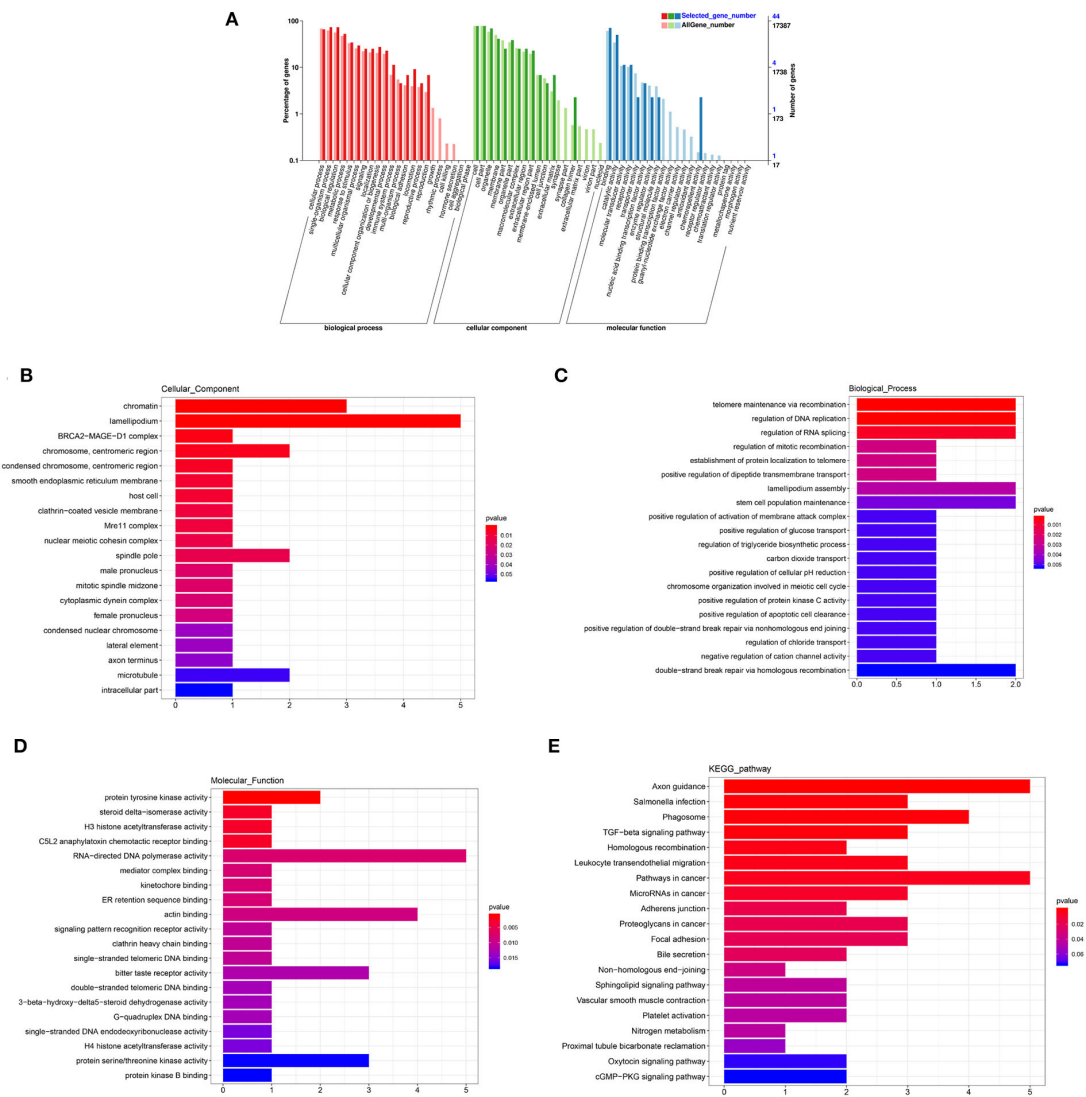
GO and KEGG analysis of DEmRNAs. **(A)** GO enrichment of all mRNAs and DEmRNAs. **(B)** Cellular component enrichment. **(C)** Biological process enrichment. **(D)** Molecular function enrichment. **(E)** KEGG analysis.

The annotated information on DEGs is shown in Supplementary Table 5. A total of 1,868 genes and 84 DEG were categorized into 56 and 38 GO functional groups, respectively (Figure 5A). GO annotation includes three categories: component category (CC), biological process (BP), and molecular function (MF). Figures 5B–D show 20 terms with the most significant differences according to *p*-value (the same as below) in each category. In CC, lamellipodium (GO: 0030027; *P* = 0.0008) contained five DEGs, chromatin (GO: 0000785; *P* = 0.0006) contained three DEGs; chromosome/centromeric region (GO: 0000775; *P* = 0.0039), spindle pole (GO: 0000922; *P* = 0.0137) and microtubule (GO: 0005874; *P* = 0.0544) contained two DEGs, respectively (Figure 5B). In BP, telomere maintenance via recombination (GO: 0000722; *P* = 0.0002), regulation of DNA replication (GO: 0006275; *P* = 0.0002), regulation of RNA splicing (GO: 0043484; *P* = 0.0006), lamellipodium assembly (GO: 0030032; *P* = 0.0033), stem cell population maintenance (GO: 0019827; *P* = 0.0046), and double-strand break repair via homologous recombination (GO: 0000724; *P* = 0.0053) contained two DEGs, respectively (Figure 5C). In MF, five DEGs were enriched in RNA-directed DNA polymerase activity (GO: 0003964; *P* = 0.0075); four DEGs were enriched in actin binding (GO: 0003779; *P* = 0.0088); bitter taste receptor activity (GO: 0033038; *P* = 0.0122) and protein serine/threonine kinase activity (GO: 0004674; *P* = 0.0182) contained three DEGs, respectively. KEGG pathways showed that (Figure 5E), besides axon guidance and pathways in cancer, there were more DEGs in phagosome (ko04145; *P* = 0.0013), focal adhesion (ko04510; *P* = 0.0199), and TGF-β signaling pathway (ko04350; *P* = 0.0017). The results of GO and KEGG indicated that these terms and pathways with more differences were likely to regulate cell proliferation, BTB dynamic, and phagocytosis in testis.

### GO and KEGG analysis of DElncRNAs

**Figure 6:**
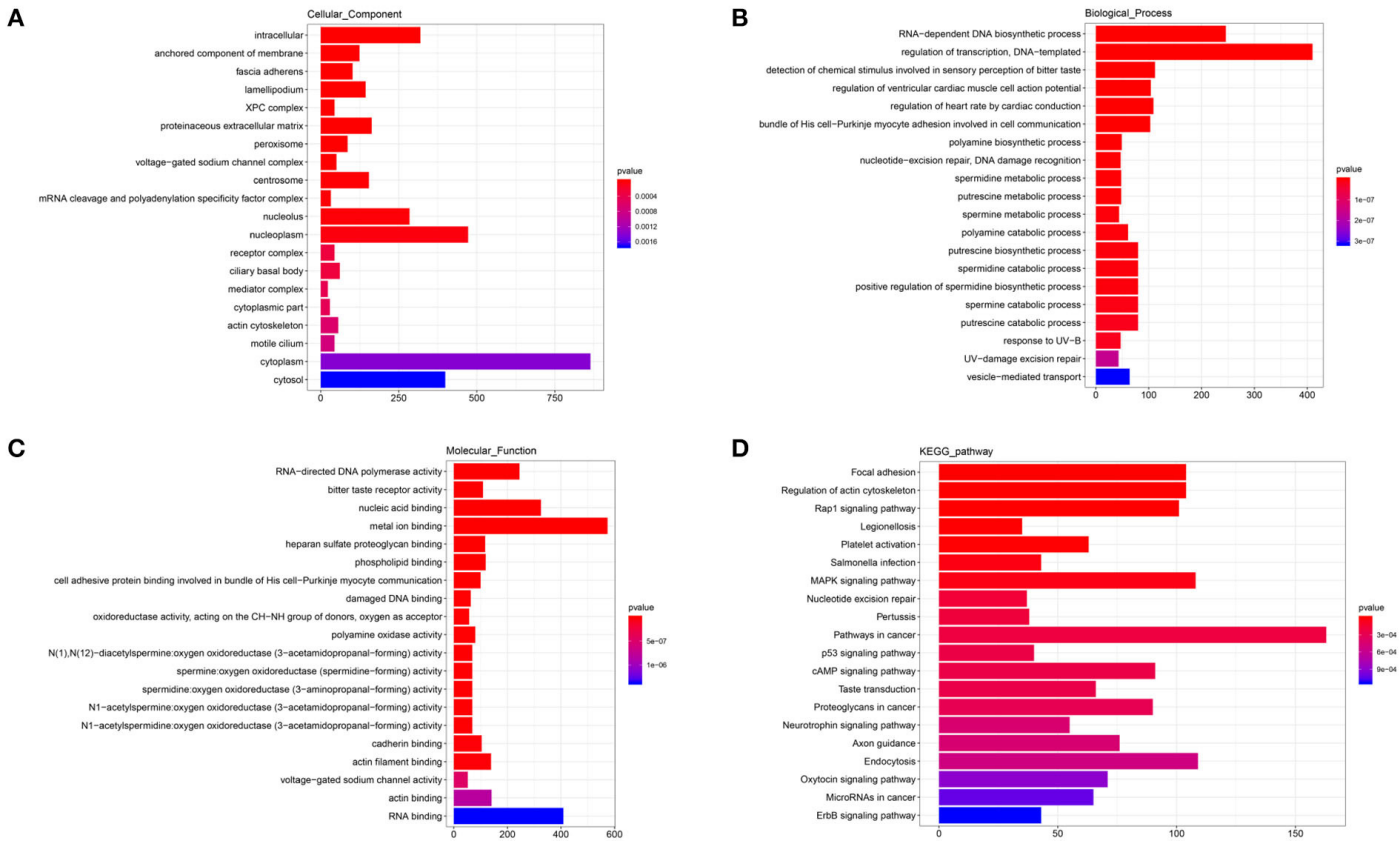
GO and KEGG analysis of DElncRNAs. **(A)** Cellular component enrichment. **(B)** Biological process enrichment. **(C)** Molecular function enrichment. **(D)** KEGG analysis.

The annotated information on target genes of DElncRNAs are shown in Supplementary Table 6. The results of GO enrichment showed that intracellular (GO: 0005622; *P* = 0.0000), nucleolus (GO: 0005730; *P* = 0.0000), nucleoplasm (GO: 0005654; *P* = 0.0000), cytoplasm (GO: 0005737; *P* = 0.0019), and cytosol (GO: 0005829; *P* = 0.0026) enriched more than 250 target genes and were significantly higher than other terms in CC category (Figure 6A). In BP, RNA-dependent DNA biosynthetic process (GO: 0006278; *P* = 0.0000) and regulation of transcription/DNA-templated (GO: 0006355; *P* = 0.0000) were two terms enriched to most target genes (Figure 6B). And in MF, RNA-directed DNA polymerase activity (GO: 0003964; *P* = 0.0000), nucleic acid binding (GO: 0003676; *P* = 0.0000), and metal ion binding (GO: 0046872; *P* = 0.0000) and RNA binding (GO: 0003723; *P* = 0.0071) enriched more than 200 target genes (Figure 6C). In the top 20 KEGG pathways, focal adhesion (ko04510; *P* = 0.0000), regulation of actin cytoskeleton (ko04810; *P* = 0.0000), Rap1 signaling pathway (ko04015; *P* = 0.0000), MAPK signaling pathway (ko04010; *P* = 0.00010), pathways in cancer (ko05200; *P* = 0.0007), and endocytosis (ko04144; *P* = 0.0003) enriched more than 100 target genes (Figure 6D). These pathways were involved in the regulation of cell proliferation and phagocytosis.

### GO and KEGG analysis of DEmiRNAs

**Figure 7:**
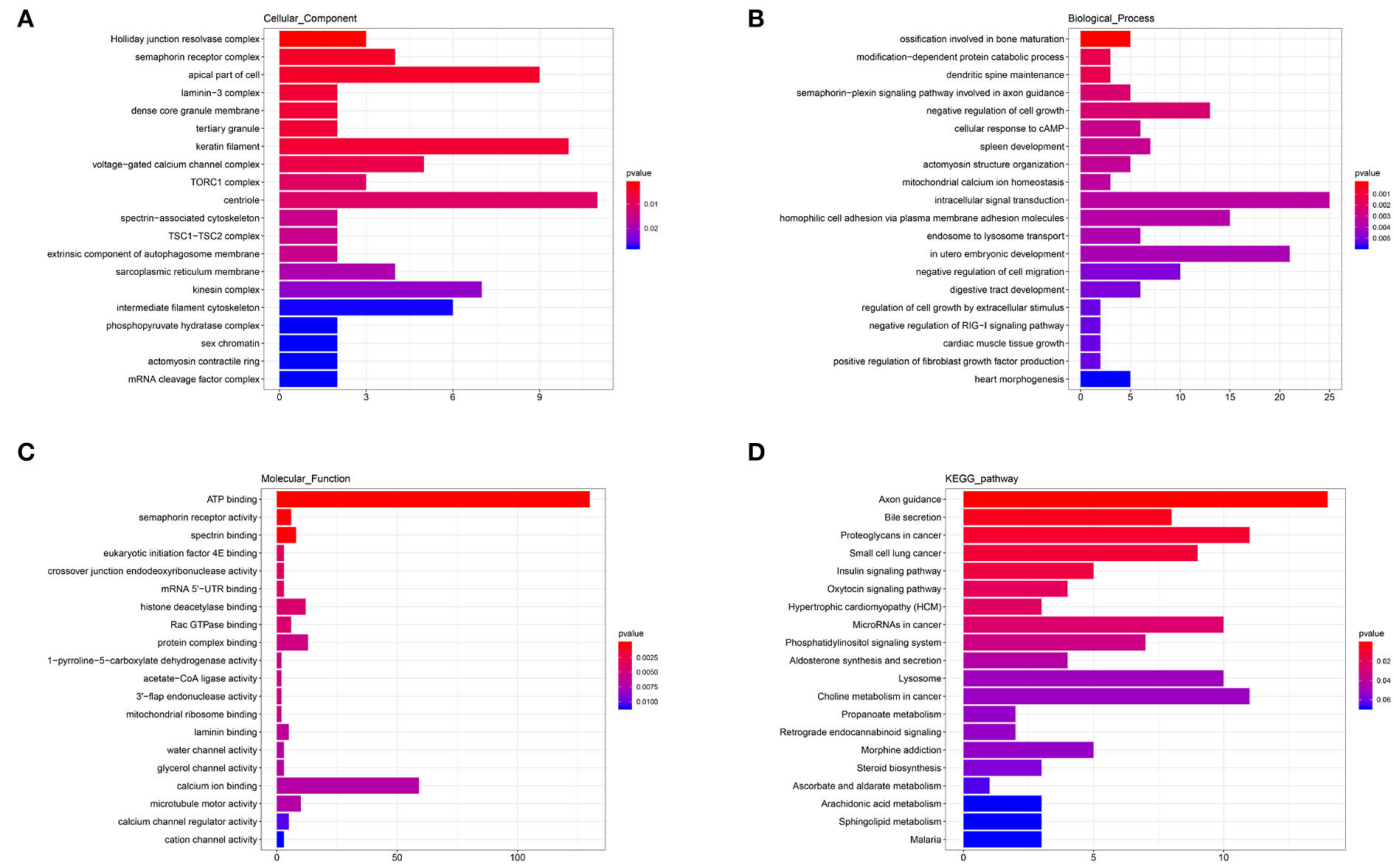
GO and KEGG analysis of DEmiRNAs. **(A)** Cellular component enrichment. **(B)** Biological process enrichment. **(C)** Molecular function enrichment. **(D)** KEGG analysis.

The annotated information on target genes of DEmiRNAs are shown in Supplementary Table 7. GO enrichment indicated that apical part of cell (GO: 0045177; *P* = 0.0040), keratin filament (GO: 0045095; *P* = 0.0051), centriole (GO: 00005814; *P* = 0.0105), and kinesin complex (GO: 0005871; *P* = 0.0219) enriched more than six target genes in CC (Figure 7A). In BP, intracellular signal transduction (GO: 0035556; *P* = 0.0034), homophilic cell adhesion *via* plasma membrane adhesion molecules (GO: 0007156; *P* = 0.0036), and *in utero* embryonic development (GO: 0001701; *P* = 0.0039) enriched no less than 15 target genes (Figure 7B). In the MF category, ATP binding (GO: 0005524; *P* = 0.0001) enriched more than 125 target genes and calcium ion binding (GO: 0005509; *P* = 0.0068) enriched more than 50 target genes, which were significantly higher than other terms (Figure 7C). KEGG showed that axon guidance (ko04360; *P* = 0.0014), proteoglycans in cancer (ko05205; *P* = 0.0093), microRNAs in cancer (ko05206; *P* = 0.0260), lysosome (ko04142; *P* = 0.0503), and choline metabolism in cancer (ko05231; *P* = 0.0513) enriched no less than 10 target genes. In addition, a total of seven target genes were enriched to aldosterone synthesis and secretion (ko04925; *P* = 0.0432) and steroid biosynthesis (ko00100; *P* = 0.0576) (Figure 7D). The results indicated that there were differences in the secretion of sterol hormones and cell metabolism.

### Interaction network of DEmRNAs-DElncRNAs-DEmiRNAs

**Figure 8:**
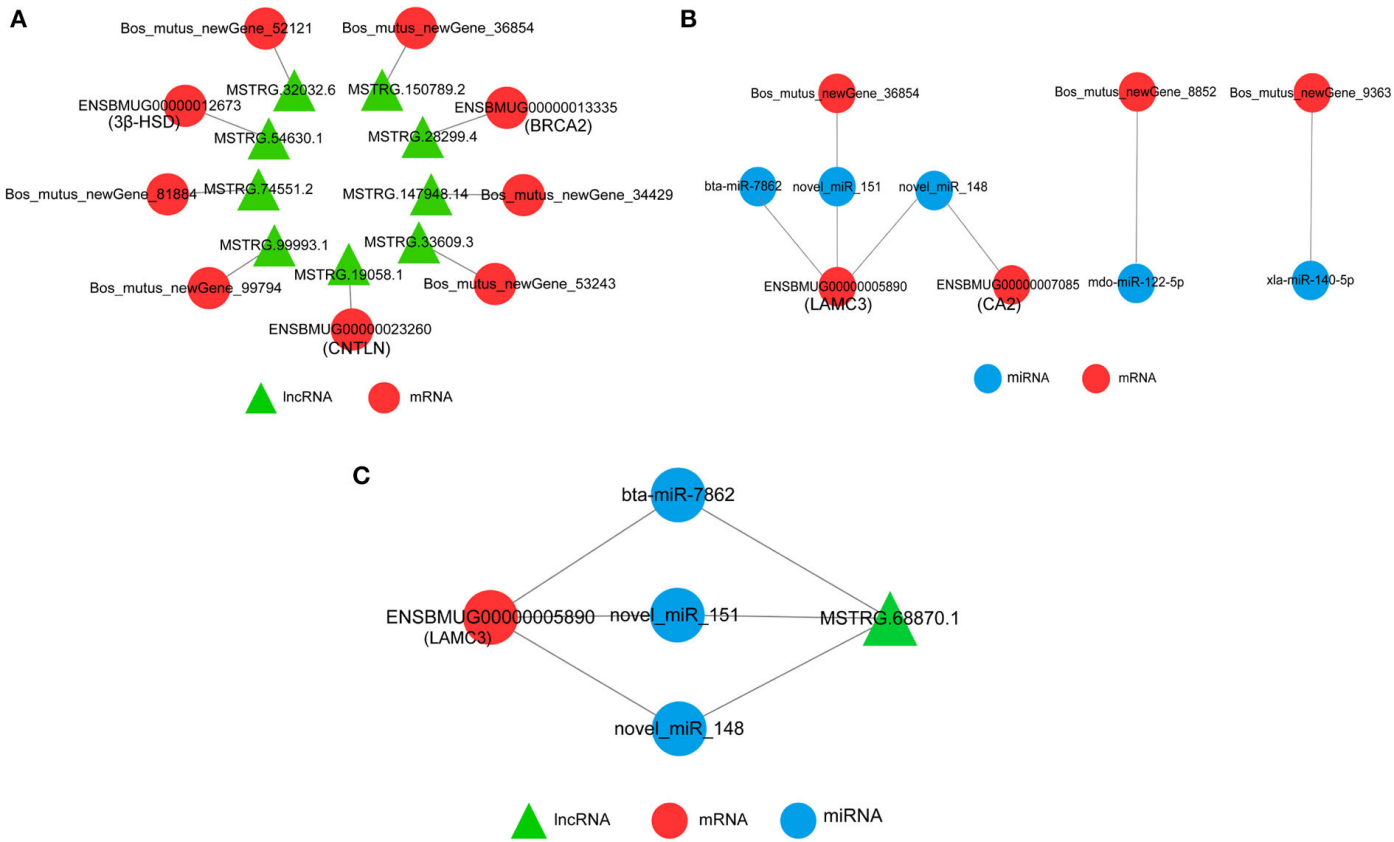
Interaction network of DEmRNAs, DElncRNAs, and DEmiRNAs. **(A)** Interaction of DEmRNAs and DElncRNAs. **(B)** Interaction of DEmRNAs and DEmiRNAs. **(C)** CeRNA network of DEmRNAs, DElncRNAs, and DEmiRNAs.

The network between DEmRNAs and DElncRNAs showed that nine DElncRNAs targeted nine DEmRNAs, among which 3β-HSD (ENSBMUG00000012673), CNTLN (ENSBMUG00000023260), and BRCA2 (ENSBMUG00000013335) were the target genes of MSTRG.54630.1, MSTRG.19058.1, and MSTRG.28299.4, respectively, while the other six DEmRNAs were new genes whose functions remained to be explored (Figure 8A). The target relationship of DEmRNAs and DEmiRNAs indicated that bta-miR-7862, novel-miR-151, and novel-miR-148 regulated LAMC3 (ENSBMUG00000005890) together; in addition, CA2 (ENSBMUG00000007085) was also a target gene of novel-miR-148. miRNAs obtained by sequencing that had the same mature sequence as other species, such as mdo-miR-122-5p and xla-miR-140-5p, are involved in the regulation of new genes (Figure 8B). Analysis of the ceRNA network showed that MSTRG.68870.1 might be a sponge of bta-miR-7862, novel-miR-151, and novel-miR-148 to regulate LAMC3 (Figure 8C).

## Discussion

It is well known that the testis is the most crucial organ for maintaining male function. With the advancement of science and technology, the regulatory role of ncRNAs is increasingly attracting attention. LCs and SCs play pivotal roles in spermatogenesis and androgen secretion. Proteins, hormones, and growth factors secreted by them regulate the testis environment altogether but the molecular mechanisms involved in these processes are poorly understood, especially the regulation of ncRNAs. In order to further study the regulatory relationship between the two types of cells, high-throughput sequencing was used in this study to explore the differences in their gene expression patterns. We used mechanical separation, enzymatic digestion, gradient centrifugation, starvation culture, and hypotonic treatment to isolate and purify yak LCs and SCs. They were identified as being of higher purity because of the positive results obtained during their specific staining. In addition, the secretion of testosterone in sub-cultured LCs was detected by ELISA, indicating that the cells' function was relatively complete. The previous experiments proved that the yak LCs and SCs obtained by primary culture could be used for sequencing and further analysis.

Sequencing technology has widely been used in animals to mine potential regulatory molecules. The ncRNAs in testis, which have been identified and functionally verified, suggest that they are involved in testicular development and sperm regulation (17–19, 33). In this study, we constructed a linear RNA library and miRNA library. After sequencing and alignment, a total of 5,767 mRNAs, 12,006 lncRNAs, and 6312 miRNAs were identified, indicating that there were a lot of non-coding regulations in testicular LCs and SCs. LincRNA accounted for 62% by our statistics, while human lincRNAs accounted for nearly half of its total lncRNAs by the analysis of the FANTOM5 consortium (34). After identification and analysis of gene expression, we clearly saw the number and feature of protein-coding genes and non-coding genes in two kinds of cells in yak testis. Further analysis of the sequencing results is very valuable for understanding the regulatory mechanism.

We obtained 84 DEGs in the two groups of testicular cells by sequencing and differential analysis. Most of these genes were enriched into three categories of GO terms which were involved in cytoskeletal activities, mitosis of cells, and the regulation of enzyme activity. This was an important signal that there was much difference in cell proliferation and cytoskeleton-related functions between LCs and SCs. SCs in mammals are a kind of special cells, which have two stages of proliferation and differentiation. From 4 weeks to 13–20 weeks in cattle, SCs enter the second proliferation cycle and become mature SCs from immature SCs. And mature SCs no longer have the ability to proliferate *in vivo*. Importantly, adult mouse and human Sertoli cells can resume proliferation *in vitro*, suggesting that they could be proliferating cells at rest rather than terminally differentiating cells (35, 36). Our study also demonstrated that Sertoli cells from sexually mature yaks restored their proliferative capacity *in vitro*. Distinct populations of LCs develop along the life span of males, giving rise to fetal, neonatal (primates), and adult LCs, they can differentiate into the mature population of LCs with the main function to provide androgens necessary for the maintenance of spermatogenesis and extra-gonadal androgen actions (37). KEGG analysis drew attention to the differences in phagocytic regulation between the two types of cells. Studies have confirmed that nonviable germinal cells degenerate during spermatogenesis and their residues are actively phagocytosed by SCs (38). The role of phagocytosis in testis interstitium is completed by macrophages, which remove senescent LCs, bacterium, and viruses that invade the testis (39).

Upon analysis, there were a total of 172 DElncRNAs, of which 136 lncRNAs were highly expressed in LCs and 36 in SCs. From the results of GO enrichment, we observed that their target genes mainly participated in the regulation of activation of biochemical enzymes and the binding of proteins/ions by influencing processes of transcription and translation. KEGG analysis results hinted that there existed differences in cell proliferation, differentiation, survival, apoptosis, and cell junctions. This indicated that lncRNA played a major role in the regulation of cell fate and function in LCs and SCs by regulating cellular processes, such as chromosome and genome modification, transcription activation and interference, and nuclear transport (40). LncRNAs also can act as ceRNAs to inhibit the function of miRNAs and bind to DNA binding proteins to inhibit their interaction with the target genes (41). Studies have shown that lncRNA MIR22HG promotes LCs apoptosis by acting as ceRNA for miRNA-125a-5p that targets NDRG2 in late-onset hypogonadism (42). In addition, LncRNA Tug1 has been shown to maintain the integrity of BTB by regulating Ccl2 in mice (43).

According to our analysis, 90 miRNAs were considered to be different between LCs and SCs. The results of GO and KEGG indicated that miRNAs differently regulated the processes of phagocytosis, cell metabolism, and hormone secretion in LCs and SCs. Recent studies have reported that miR-9-3p, from SC-derived exosomes, has been verified to suppress testosterone biosynthesis of LCs by targeting STAR (44). miR-471-5p transgenic mice showed increased GCs apoptosis and compromised male fertility, which was caused by defective engulfment and impaired LAP-mediated clearance of apoptotic GCs. Overexpression of miR-471-5p downregulated Dock180 which interacts with autophagy member proteins to constitute a functional LC3-dependent phagocytic complex. Furthermore, miR-471-5p plays an important role in regulating GCs attachment to SCs as well as BTB integrity, a well-established androgen-dependent event (45).

Network interaction analysis showed that 3β-HSD, CNTLN, and BRCA2 might be regulated by lncRNAs. 3β-HSD is one of the key enzymes in the biosynthesis of androgens, and its activity in the testes is essential for normal steroidogenesis, including testosterone production in LCs, and subsequently for reproduction (46). Though 3β-HSD was detected in SCs in some primate species, the predominant site of 3β-HSD expression in the testis is in LCs (47). In this study, 3β-HSD mRNA in SCs was higher than that in LCs, whether miRNA inhibited the translation process of 3β-HSD in SCs needs further study. CNTLN is localized to the proximal ends of centrioles in somatic cells and is required for centrosome cohesion by mediating interaction between C-Nap1 and Cep6837 (48). Cytokinesis is a highly controlled process that involves numerous proteins. CNTLN facilitates equal segregation of cytoplasmic contents between daughter cells by recruiting the endosomal sorting complex required for transport machinery at the midbody (49). During sperm production, CNTLN, cooperating with SUN5 and PMFBP1, participates in HTCA assembly and integration of sperm head to the tail (50). In our results, the higher mRNA level in LCs indicated that cell proliferation was more obvious in them. BRCA2 is a tumor suppressor and a key mediator of homologous recombination (HR) in vertebrates. Repair of DNA damage through homologous recombination requires BRCA2 to form complexes with MEILB2 and PP2A-B56 (51, 52). A study by Brandsma revealed that BRCA2 formed direct and evolutionarily conserved interaction with HSF2BP, which is required for mouse spermatogenesis (53). In our results, BRCA2 mRNA was detected in both types of cells, and the expression level was higher in LCs, indicating that there was a higher level of homologous recombination in LC, which was conducive to cell proliferation and renewal. CAs are a group of isozymes that differ in kinetic properties, inhibitor sensitivity, and selectivity, and are essential for mitochondrial biogenesis, glucose, and lipid metabolism in human Sertoli cells (54). CA2 and CA4 play a role in acidifying the capacity of cells, their regulation of pH is crucial for sperm storage and movement, as well as capacitation during fertilization (55). Therefore, considering previous reports and our findings that the CA2 mRNA level in SCs was higher than that in LCs, novel-miR-148 may regulate pH and energy metabolism of SCs by affecting the function of CA2. LAMC3 is a ligand, which usually works in concert with integrin receptors through interaction between the extracellular matrix and epithelial cells mediated *via* their C-terminal region. The C-terminal region then interacts with a corresponding integrin receptor, which in turn induces integrin-based signaling to modulate multiple cellular functions (56). A study on testis indicated that LAMC3, at the SCs-spermatid interacting site including apical ectoplasmic specialization and basement membrane, release biologically active peptides, such as F5-peptide, which is capable of perturbing SCs BTB function and apical ectoplasmic specialization adhesion function, thereby promoting the transport of preleptotene spermatocytes across immunological barriers near the basement membrane and the release of fully developed spermatids near the tubule lumen (57). ceRNA network analysis indicated that LAMC3 may be regulated by MSTRG.68870, which acts as a sponge of bta-miR-7862, novel-miR-151, and novel-miR-148. We also noted that many new genes and novel miRNAs are involved in the network regulation of LCs and SCs, suggesting that there is still a huge space for exploration in the regulation of LCs and SCs on testicular function.

## Conclusion

In this study, differential expression of mRNAs, lncRNAs, and miRNAs between LCs and SCs of yak and the construction of the differential regulation network of ncRNAs were reported for the first time. The differences between the two cellular physiological processes are mainly reflected in steroid hormone synthesis, cell proliferation and metabolism, and BTB dynamic regulation. Furthermore, 3β-HSD, CNTLN, BRCA2 may be key factors to maintain testosterone secretion function and cell proliferation capacity in LCs, while CA2 and LAMC3 play major roles regulated by ncRNAs in cell metabolism and BTB dynamic in SCs. In conclusion, this study provides basic research direction to explore key molecules of LCs and SCs function affected by ncRNAs.

## Data availability statement

The datasets presented in this study can be found in online repositories. The names of the repository/repositories and accession number(s) can be found in the article/Supplementary material.

## Ethics statement

The animal study was reviewed and approved by the College of Veterinary Medicine of Gansu Agricultural University (Ethic approval file No. GSAU-Eth-VMC-2022-19).

## Author contributions

YP, MW, and YW conceived and designed the study. ML, JW, and TZhan took yak testis samples. YW, ML, and XH cultured LCs and SCs. YW, LZ, and TZhao analyzed the data. YW and TD uploaded the raw data to NCBI. YW wrote the manuscript. YW, SA, YC, and SY contributed to the revisions of the manuscript. All authors have read and approved the final version of the manuscript.

## Funding

This study was supported by the National Natural Science Foundation of China (32160859), the National Natural Science Foundation of China (Surface Project) (31972760), the Major Science and Technology Projects of Gansu Province (21ZD10NA001), and the Key Talent Project of Gansu Province: Technology Research and Demonstration of High-efficiency-Health-Ecological of Characteristic Livestock in Gansu Agro-pastoral Ecotone.

## Conflict of interest

The authors declare that the research was conducted in the absence of any commercial or financial relationships that could be construed as a potential conflict of interest.

## Publisher's note

All claims expressed in this article are solely those of the authors and do not necessarily represent those of their affiliated organizations, or those of the publisher, the editors and the reviewers. Any product that may be evaluated in this article, or claim that may be made by its manufacturer, is not guaranteed or endorsed by the publisher.
