# Supplementary material for: Transcriptome sequencing reveals differences between leydig cells and sertoli cells of yak
Source: Front Vet Sci. 2022 Aug 24;9:960250. doi: 10.3389/fvets.2022.960250 (PMC9449347; doi:10.3389/fvets.2022.960250)
Supplement: Supplementary file 1 [file Table_1.DOCX]

**Supplementary Material 1: the primers sequence of RT-qPCR**

| Gene | Primer sequence | annealing temperature(℃) | Product length(bp) |
| --- | --- | --- | --- |
| 3β-HSD | F: CAGTCTATGTTGGCAATGTG | 55 | 113 |
|  | R: CGTGTCGTCTGAGATGTAG |  |  |
| ROCK2 | F: ATCTCATCTGTGCCTTCTTA | 55 | 167 |
|  | R: GCTGCTGTCTATGTCACT |  |  |
| ROCK1 | F: ATGAGAAGAGGAGGAATGTAG | 55 | 173 |
|  | R: TCTAACCGCTGTGTCTGA |  |  |
| LAMC3 | F: TCTTGGAGCAGGTGACAGGACT | 60 | 190 |
|  | R: AGAGATGCGAGGATGGTGGCT |  |  |
| β-ACTIN | F: CGTCCGTGACATCAAGGAGAAGC | 50-60 | 108 |
|  | R: GGAACCGCTCATTGCCGATGG |  |  |
| MSTRG.129128.1 | F: ATATCCGCAGCAGGTCTCC | 60 | 125 |
|  | R: GTCCGCACCAGTTCTAAGTC |  |  |
| MSTRG.30014.1 | F: TTAGCCTGAAGAACGGTATT | 50 | 191 |
|  | R: CACAAGTAAGGAACCAGAGA |  |  |
| MSTRG.9090.10 | F: TCTCTTACCTGTAACCTGTG | 50 | 102 |
|  | R: TTCTCTGTGCTGGCTCTA |  |  |
| MSTRG.4865.1 | F: TGGCTCACTGGTAAAGAATCTG | 52 | 110 |
|  | R: AGGCAAGACACTGGAATGGA |  |  |
| MSTRG.123492.1 | F: GCAAGTGGAGGAAGCAGTGAAGT | 56 | 101 |
|  | R: AGCACCGTCCAATCTCTGTCAAC |  |  |
| MSTRG.68870.1 | F: TCCTCTGCCACCTGCTGTCTT | 55 | 167 |
|  | R: CGTTGCCGTGCTGAGTGCTAT |  |  |
| bta-miR-7862 | TGGTGCTCCCTGGAGCT | 60 |  |
| bta-miR-122 | CCGTGGAGTGTGACAATGGTGTTTG | 60 |  |
| novel-miR-148 | TGGTGCTCCCTGGAGCT | 60 |  |
| xla-miR-140-5p | GCCAGTGGTTTTACCCTATGGTAGG |  |  |
| bta-miR-3431 | CCTCAGTCAGCCTTGTGGATGT | 60 |  |
| bta-miR-2307 | CCAGTGATGATGAACCCTCTGAGC | 60 |  |
| novel-miR-151 | TATATATAGCGCCCGGAGTGTGGC | 60 |  |
| U6 | F: GGAACGATACAGAGAAGATTAGC | 60 |  |
|  | R: TGGAACGCTTCACGAATTTGCG |  |  |
